# Poplar's Measures of Disinfection

**Published:** 1919-08-23

**Authors:** 


### Poplar's Measures of Disinfection.

In a voluminous report, Dr. Alexander, the medical officer of health, gives particulars of the public health of the Metropolitan Borough of Poplar during the year 1917. The estimated population of the borough for 1917 was 143,443, compared with the 1911 census population of 162,442. In common with other districts throughout the country the birth-rate for 1917 showed a marked fall, being 23.13 compared with 26.92 for 1916, but it is satisfactory to note the very considerable fall in the infant mortality, the death-rate under one year per 1,000 births being 95.13, compared with 103.89 and 133.98 for the two previous years. There were 2,812 deaths registered in the borough during the year, giving a death-rate of 16.84 compared with 16.27 for the previous year. The zymotic death-rate shows a slight increase from 1.53 to 1.76. A rise is recorded in the death-rate from respiratory diseases, the rate being 3.78 compared with 3.55 for the previous year. The death-rate from tuberculosis has increased since 1915, the rate of 2.34 being the highest recorded since 1908. Various particulars of the notifications received and of the deaths from tuberculosis in the various districts are given in a number of tables. Dr. Alexander gives some interesting information in his report as to the measures adopted by his Council to secure efficient disinfection and cleansing. There are seven depôts for the distribution of electrolytic disinfecting fluid, which continues to be in great demand by the public. During the year, 55,800 gallons of this fluid were distributed, and 4,238 children belonging to the borough and 645 children outside the borough were bathed and had their clothes disinfected. No case of smallpox, typhus fever, plague, or anthrax was notified during the year. Fifteen cases of puerperal fever were notified, of which number two died. Eight cases of cerebro-spinal fever occurred, and there were six deaths from this cause. The question of the provision of houses for the working classes came up for consideration during the year, and it was decided that it was undesirable for the Council to embark upon a housing scheme for the borough, but that the London County Council, as the housing authority for London, should prepare a scheme for the whole of London. Dr. Alexander's report gives much valuable information, but its value would be increased if a brief summary of its main features were appended.

